# Pneumothorax in neuromuscular disease associated with lung volume recruitment and mechanical insufflation‐exsufflation

**DOI:** 10.1002/rcr2.447

**Published:** 2019-06-13

**Authors:** Luke Andrew McDonald, David John Berlowitz, Mark Erskine Howard, Linda Rautela, Caroline Chao, Nicole Sheers

**Affiliations:** ^1^ Department of Physiotherapy Austin Health Heidelberg Victoria Australia; ^2^ Department of Respiratory and Sleep Medicine Austin Health Heidelberg Victoria Australia; ^3^ Institute for Breathing and Sleep Heidelberg Victoria Australia; ^4^ Department of Physiotherapy The University of Melbourne Parkville Victoria Australia

**Keywords:** Lung volume recruitment, neuromuscular disease, physiotherapy, pneumothorax, respiratory therapy

## Abstract

A 25‐year‐old male with Duchenne muscular dystrophy and a 73‐year‐old male with motor neurone disease both presented with chest pain and increasing dyspnoea following routine mechanical insufflation‐exsufflation or lung volume recruitment, on a background of long‐term non‐invasive ventilation. In each case, chest radiograph revealed a pneumothorax. In both cases the pneumothorax fully resolved following insertion of an intercostal catheter. There was no immediate recurrence and the patients were discharged home and ceased ongoing prophylactic respiratory therapy, although one person had recurrent pneumothoraces subsequently. This rare but serious complication highlights the need for careful risk/benefit analysis by clinicians prescribing these therapies.

**This case report is an illustration of an invited review available in Respirology:** Sheers, N, Howard, ME, Berlowitz, DJ. Respiratory adjuncts to NIV in neuromuscular disease. *Respirology*. 2019; 24: 512– 520. https://doi.org/10.1111/resp.13431

## Introduction

Neuromuscular disease (NMD) often involves muscles of the respiratory system, and may result in ventilatory failure and increased risk of respiratory tract infection 1, 2. Mechanical ventilatory support, such as non‐invasive ventilation (NIV) is the primary respiratory therapy for patients with NMD and respiratory failure; however, clinical adjuncts focusing on cough augmentation, lung inflation, and chest wall mobility are frequently used both in hospital and the community 1. Common examples of these adjunctive therapies are lung volume recruitment (LVR) and mechanical insufflation‐exsufflation (MI‐E). MI‐E uses a dedicated device for delivering assisted inspiration at a set pressure followed by a rapid cycle to exsufflation at negative pressure. LVR can be performed using a manual resuscitation bag with or without a one‐way valve, glossopharyngeal breathing or the inspiratory phase of a (typically) volume cycled NIV device 1, 3. Because both MI‐E and LVR increase inspiratory capacity above a maximal spontaneous inspiration, it is important to consider the risk of pneumothorax or other overexpansion injury when prescribing these techniques.

This report describes two cases of pneumothorax associated with LVR and MI‐E techniques.

## Case Report

Case 1: A 25‐year‐old male with Duchenne muscular dystrophy and baseline respiratory function as per Table 1, using nocturnal NIV and mouthpiece intermittent positive pressure ventilation (MIPPV) presented to the emergency department with right‐sided chest pain and dyspnoea. The evening prior to onset of symptoms, he reported using MIPPV for 10 h. He then completed five cycles of MI‐E (pressures +50 cm of water (cmH_2_O) for insufflation, −47 cmH_2_O for exsufflation) to clear excess saliva. He went to bed on NIV via total face mask (settings per Table 2) and upon waking four hours later, noticed sharp right‐sided chest pain. The patient completed another five cycles of MI‐E; however, this worsened his symptoms, prompting emergency department presentation.

**Table 1:** Respiratory function.

	Case 1	Case 2
Vital capacity	0.420 L (9%)	2.828 L (71%)
Peak cough flow	91 L/min	310 L/min
Functional residual capacity	1.13 L (38%)	1.94 L (53%)
Total lung capacity	1.36 L (22%)	4.48 L (64%)
Residual volume	0.94 L (63%)	1.58 L (60%)
Inspiratory capacity	0.23 L	2.55 L (83%)
Average tidal volume	0.21 L	0.765 L
Total respiratory system compliance	0.0167 L/cmH_2_O	0.0851 L/cmH_2_O

**Table 2:** Values are presented as absolute measurements (percentage of predicted normal, where available; static lung volume measurements were obtained via the nitrogen washout technique).

Ventilator settings	Case 1	Case 2
NIV settings	Nocturnal: mode ST; IPAP 23; EPAP 11; backup rate 18	Nocturnal: mode ST; IPAP 13; EPAP 8; backup rate 12
Machine: ResMed VPAP IV	Interface: total face mask	Interface: full face mask
MIPPV settings	Daytime: mode ACV	NA
Machine: ResMed VSIII	VT 0.500 L; Ti 1.4; Backup rate 5	

ACV, assist control ventilation; EPAP, expiratory positive airway pressure; IPAP, inspiratory positive airway pressure; MIPPV, mouthpiece intermittent positive pressure ventilation; NIV, non‐invasive ventilation; ST, spontaneous‐timed; Ti, inspiratory time; VT, tidal volume. ResMed VPAP IV and ResMed VSIII (ResMed, Bella Vista, Australia).

**Figure 1:**
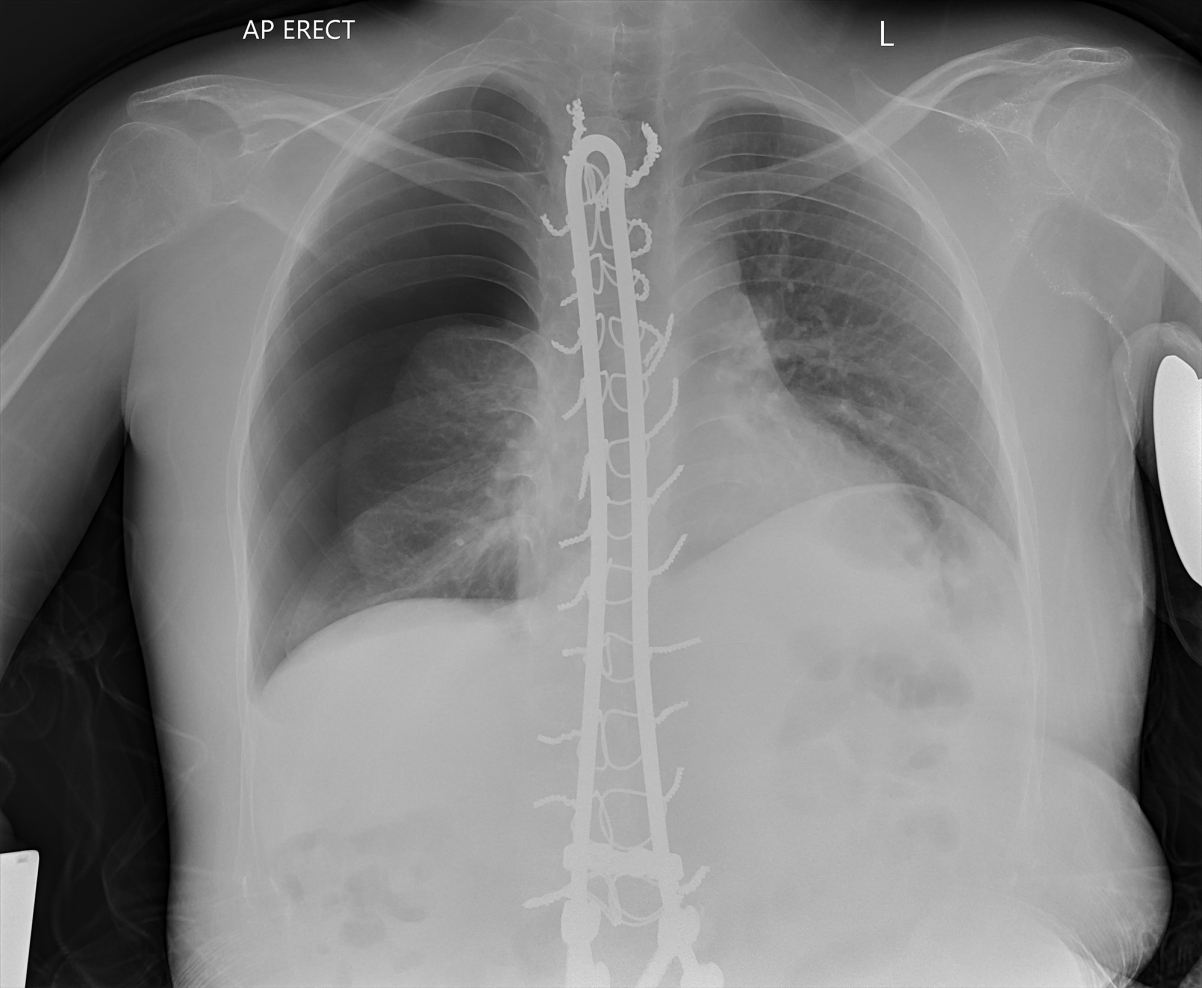
Right‐sided pneumothorax (Case 1).

On presentation, the patient was tachypnoeic with a respiratory rate of 36 breaths/min. All other vital signs, including pulse oximetry were within normal limits. Initial arterial blood gases were unremarkable. Chest radiograph performed in the emergency department revealed a large right‐sided pneumothorax (Fig. 1) and a pigtail intercostal catheter (ICC) was inserted and placed on −10 cmH_2_O suction. Repeat chest radiograph two hours later demonstrated poor re‐expansion of the right lung. The suction was increased to −20 cmH_2_O, resulting in good re‐expansion on subsequent imaging. The patient used NIV on usual settings continuously throughout this period. The ICC was removed on the third day of admission and the patient was discharged on day five with advice to cease all MI‐E until clinical review in two weeks.

The MI‐E pressures were decreased to +25 cmH_2_O insufflation and −40 cmH_2_O exsufflation by a physiotherapist at this review, with instructions to use sparingly for cough augmentation or saliva clearance only. Pharmacological modalities of saliva management were recommended; however, the patient had no success with these previously.

Unfortunately, this patient represented on three occasions (total of four presentations) with right‐sided pneumothorax in the following six months. Bedside chemical pleurodesis was conducted on two occasions (iodine and alcohol/iodine, respectively), both failing to prevent recurrence. The patient was discharged home with a long‐term pleural catheter after the fourth admission, which was removed two months later. The patient continues to use MI‐E sparingly and has had no subsequent recurrence.

Case 2: A 71‐year‐old male with motor neurone disease presented with a 36‐h history of worsening dyspnoea that began immediately after LVR therapy. This patient did not use LVR routinely, instead using as required for cough augmentation. He noted a sharp, sudden onset of central chest pain following LVR, but attributed this to musculoskeletal stretching of his thoracic cage. He described mild “shallow breathing” that worsened over the next two nights (despite using nocturnal NIV with usual settings; Table 2) and subsequently presented to the emergency department. Chest radiograph revealed a large right‐sided pneumothorax; the apex of the right lung projected over the inferior margin of the sixth rib, with no mediastinal displacement. An ICC was inserted, with resolution of the pneumothorax and removal of the ICC occurring on the fourth day of admission. There was no recurrence with resumption of use of NIV. He was discharged with advice to cease LVR.

## Discussion

LVR and MI‐E are therapy modalities frequently recommended as part of the respiratory management in those with NMD. While the clinical utility of these techniques has been established during episodes of respiratory illness 4, the benefits of prophylactic use remains unclear 5. As such, consideration of the risk/benefit profile for individuals using these therapies is important. When considering risk, the available literature suggests that pneumothorax associated with MI‐E or LVR, while rare, is a known and potentially life‐threatening complication. In 2013, Westermann et al. published a case of pneumothorax following LVR, in which the patient required emergency intubation and spent 20 days in hospital following drainage and pleurodesis 6. Suri et al. reported two cases of pneumothorax associated with MI‐E in patients with NMD, where one of the patients died due to cardiovascular collapse on re‐expansion of the affected lung 7. More recently Loewen et al. presented a case series of six patients with pneumothorax in patients on home mechanical ventilation (HMV). In addition to HMV, all patients used LVR or MI‐E 8. While these published cases of pneumothorax associated with MI‐E/LVR in the NMD population are clearly associated with serious outcomes, the event rate is likely very low. A study from 2013 conducted by Garner et al. estimated a minimum prevalence of HMV usage in Australia and New Zealand of 3.0 people per 100,000 9. The apparent scarcity of pneumothorax within an already rare population further supports the contention that the overall event rate of pneumothorax is very low.

The decision to cease prophylactic MI‐E in the patient in Case 1 was driven by the belief that the risk of further pneumothoraces outweighed any potential benefits of continuing therapy. It is not certain that the first episode of pneumothorax was caused by the use of MI‐E, as the session was largely unremarkable with no acute onset of symptoms. It is possible that this event was related to the combined use of MIPPV, NIV, and MI‐E. Nonetheless, given the recent pneumothorax, the risk of recurrence was considered high and the use of MI‐E for saliva clearance deemed excessive. Despite having significantly impaired lung function (Table 1), his respiratory symptoms were well managed by nocturnal and daytime NIV and he did not have a history of recurrent respiratory tract infections that may warrant routine MI‐E use.

In Case 2, the relationship between LVR and the pneumothorax appeared causative. The patient described sharp chest pain immediately after the LVR session and progressive dyspnoea. Given that this patient did not have any known pulmonary pathologies, it is suspected that over‐expansion of alveolar units resulted in rupture and pneumothorax. Again, LVR was ceased as the patient did not have excessive pulmonary secretions. Additionally, he had relative preservation of peak cough flow, only mild ventilatory restriction (Table 1) and his nocturnal hypoventilation was well managed with NIV.

There is no published data identifying lung function thresholds or respiratory system compliance values for which the risk of pneumothorax secondary to MI‐E or LVR increases. Given the rarity of this complication, it is unlikely that robust measures for risk of pneumothorax will be developed and instead clinicians will be required to make a judgement based on the patient's primary pathology, comorbidities 6, disease trajectory, and ability to perform the techniques safely 10. Measuring the maximum inspiratory pressure reached during prescription of inflation therapy and ensuring the patient can remove the interface instantaneously may inform safety considerations.

The presence of prior pathology is a precaution that warrants careful consideration when prescribing MI‐E or LVR prophylactically. However, even in cases where no established risk factors exist, clinicians still need to consider the goals of therapy and educate patients on the risk versus benefit.

### Disclosure Statements

Appropriate written informed consent was obtained for publication of this case report and accompanying images.
